# Tracking temporal shifts of peripheral blood NLR, PLR, and ALB: a prognostic tool for PD-1 inhibitor efficacy in advanced malignant melanoma

**DOI:** 10.3389/fonc.2025.1704359

**Published:** 2026-01-05

**Authors:** Yushu Deng, Yikai Han, Xiangrui Meng, Jiaxin Pei, Mengmeng Yang, Ziyi Xiao, Feng Wang, Taiying Lu

**Affiliations:** Department of Oncology, The First Affiliated Hospital of Zhengzhou University, Zhengzhou, Henan, China

**Keywords:** hematological markers, immunotherapy, malignant melanoma, nutritional markers, prognostic role

## Abstract

**Objective:**

PD-1 monoclonal antibodies are cornerstone therapies for advanced malignant melanoma, yet treatment response varies greatly between patients. This study investigated temporal changes in peripheral blood inflammatory and nutritional parameters during PD-1 therapy, examined their associations with clinical outcomes, and identified prognostic biomarkers.

**Methods:**

A retrospective analysis was conducted on 99 patients with advanced malignant melanoma who received PD-1 monoclonal antibody treatment at the First Affiliated Hospital of Zhengzhou University (January 2019–September 2024). Imaging evaluations (CT/MRI, with PET-CT for suspected distant metastasis) were performed at baseline (T0, before treatment), the end of the 2nd cycle (T2), and the end of the 4th cycle (T4) to assess treatment response per the immune-related Response Evaluation Criteria in Solid Tumors (irRECIST).After four treatment cycles, patients were stratified into a clinical benefit group (complete response [CR] + partial response [PR] + stable disease [SD], n=68) and a non-benefit group (progressive disease [PD], n=31) based on the immune-related Response Evaluation Criteria in Solid Tumors (irRECIST). Peripheral blood samples were collected at five time points: baseline (T0), post-first cycle (T1), post-second cycle (T2), post-third cycle (T3), and post-fourth cycle (T4). Dynamic changes in neutrophil-to-lymphocyte ratio (NLR), platelet-to-lymphocyte ratio (PLR), lymphocyte-to-monocyte ratio (LMR), systemic immune-inflammation index (SII), serum albumin (ALB), and prognostic nutritional index (PNI) were compared between groups. Line graphs and box plots were used to visualize indicator trends, while logistic regression and receiver operating characteristic (ROC) curves were applied to evaluate prognostic value.

**Results:**

Non-benefit patients showed NLR peaks at T2 and PLR peaks at T1, while benefit patients had stable levels. ALB and PNI were higher and stable in the benefit group (P<0.05). A model combining T1PLR, T1ALB, and T2NLR achieved an AUC of 0.89 (sensitivity 0.90, specificity 0.79).

**Conclusion:**

Dynamic monitoring of peripheral blood NLR, PLR, and ALB provides critical insights for predicting PD-1 immunotherapy efficacy in patients with advanced malignant melanoma. These indicators hold promise as potential clinical biomarkers to guide the development of individualized treatment strategies.

## 1 Introduction

Malignant melanoma is an aggressive cancer originating from melanocytes, known for its highly invasive biological behavior (1). From 1990 to 2021, global melanoma incidence surged by 182.3% (from 107,400 to 303,100 cases), leading to an 86.1% increase in absolute deaths (from 33,080 to 61,550 cases) (2). In 2020, there were 325,000 new cases and 57,000 deaths worldwide, with projections of 510,000 new cases and 96,000 deaths by 2040 if current incidence trends persist (3). A significant proportion of these cases present as advanced or metastatic disease at diagnosis (4), specifically unresectable Stage III or Stage IV per the 8th edition of the AJCC Melanoma Staging Guidelines where the prognosis remains particularly dismal due to limited treatment options and high metastatic potential (5). Prior to the advent of targeted and immunotherapies, advanced melanoma was nearly incurable with an overall of 5%-10% (6).

Immune checkpoint inhibitors (ICIs) (7), especially those targeting the PD-1/PD-L1 pathway, have revolutionized the treatment of advanced melanoma by effectively blocking critical immune escape mechanisms within the tumor microenvironment thus restoring the antitumor activity of T cells (8). Compared to conventional therapies, immunotherapy has significantly improved the survival of patients with advanced disease: the 5-year survival rate has jumped from <10% in the pre-ICI era to 30%–40% and the annual death rate among melanoma patients has decreased by 1.4% per year between 2017 and 2022 (9, 10).The approval of ipilimumab by the FDA in 2011 marked a turning point in the management of advanced melanoma (11). Since then, several ICIs (12), including ipilimumab, nivolumab, and pembrolizumab, have been developed and approved for treating advanced-stage melanoma (13–15). However, the therapeutic efficacy of ICIs varies widely among patients, primarily due to tumor heterogeneity and resistance mechanisms to PD-1/PD-L1 blockade (16). Clinical trials have shown that the objective response rate (ORR) for ICI monotherapy is relatively low, ranging from 20% to 45% (17–20), while combination therapies have achieved modest improvements, with ORRs still below 60% (21–23). As a result, a significant proportion of patients with advanced melanoma fail to derive meaningful clinical benefit from these therapies. One of the most pressing challenges in this field is the identification of reliable and accessible biomarkers to predict which patients are most likely to benefit from immunotherapy, either before treatment initiation or during its early phases. Addressing this unmet need could greatly enhance the precision and effectiveness of immunotherapy and improve outcomes for patients with advanced melanoma.

In recent years, hematological markers have attracted significant attention in immunotherapy research due to their convenience and non-invasive nature. Inflammatory blood markers, such as the neutrophil-to-lymphocyte ratio (NLR) (24), platelet-to-lymphocyte ratio (PLR) (25), and lactate dehydrogenase (LDH) levels (26) have shown potential in predicting the efficacy of immunotherapy in patients with malignant melanoma. A meta-analysis focusing on the Chinese population revealed that NLR (27, 28), PLR (29), and lymphocyte-to-monocyte ratio (LMR) (30) are reliable predictors of immunotherapy outcomes in malignant melanoma. These hematological markers reflect the systemic inflammatory state and immune status within the tumor microenvironment, which may help explain their prognostic capability. However, current research results vary significantly, and most studies are retrospective, lacking robust evidence from high-quality prospective trials. This limitation has hindered the widespread clinical application of these biomarkers. Moreover, single-timepoint measurements of these markers are influenced by various confounding factors, making it difficult to accurately capture the inflammatory state of patients undergoing immunotherapy. Continuous monitoring of these markers over time might provide a more accurate representation of the systemic inflammatory changes following immunotherapy. In addition to inflammatory markers, nutritional hematological indicators, such as serum albumin (31) and prognostic nutritional index (PNI) (32), have also been reported to correlate with treatment response and prognosis in patients with non-small-cell lung cancer (33)and gastric cancer (34) undergoing immunotherapy.

This study aims to retrospectively analyze the dynamic changes in hematological and nutritional markers in patients with advanced malignant melanoma undergoing PD-1-based immunotherapy. Specifically, it seeks to explore their potential role in predicting treatment responses, evaluating clinical outcomes, and monitoring the effectiveness of immunotherapy. By assessing the prognostic capability of these markers, the study aims to identify effective biomarkers that can optimize therapeutic strategies for malignant melanoma.

## 2 Method

### 2.1 Study design and inclusion/exclusion criteria

This study retrospectively collected and analyzed clinical data from patients with histopathologically confirmed unresectable stage III or IV malignant melanoma who underwent PD-1 immune checkpoint inhibitor therapy at the First Affiliated Hospital of Zhengzhou University between January 1, 2019, and September 1, 2024. The inclusion criteria were as follows:

a. Pathological Diagnosis and Staging: Histopathologically confirmed malignant melanoma, staged as advanced (unresectable stage III or stage IV) according to the 8th edition of the AJCC staging guidelines.

b. Treatment Protocols: All patients received PD-1 inhibitor-based regimens (≥4 consecutive cycles) in strict adherence to the 2022 Chinese Society of Clinical Oncology (CSCO) Guidelines for Malignant Melanoma, tailored to tumor subtype, tumor burden, and clinical characteristics. Patients were stratified into three core groups:

PD-1 Monotherapy (n=39): For patients with low tumor burden and good performance status (ECOG ≤2).

Agents: Pembrolizumab (200 mg IV q3w) or toripalimab (240 mg IV q2w).

Chemo-Immunotherapy (n=41): For patients with high tumor burden or rapid progression (aligned with guideline’s “chemotherapy-immunotherapy synergy” principle).

Agents: Toripalimab (240 mg IV q2w) + Dacarbazine 250 mg/m² IV d1-5+ cisplatin/lobaplatin (70mg/m² IV d1/30 mg/m² IV d1-3); Toripalimab (200 mg IV q2w) +Albumin-bound paclitaxel 260mg/m² IV d1+ carboplatin(AUC = 5).

Targeted-Immunotherapy (n=19).

For patients with vascular-rich tumors (guideline-recommended “immuno-anti-angiogenesis” strategy):

Agents: Camrelizumab (200 mg IV q2w) + Apatinib (500 mg PO qd, continuous); Toripalimab (240 mg IV q2w) + Apatinib (500 mg PO qd, continuous).

All regimens were determined by a multidisciplinary team, considering guideline-adapted populations, performance status, organ function, and Ki-67 index.

c. Imaging Evaluation: Baseline imaging assessment was conducted within 1 week prior to treatment initiation (T0) to establish the initial tumor burden. Adherence to regular imaging follow-up was a critical inclusion criterion, defined as standardized imaging assessments performed at two predefined post-treatment time points: the end of the 2nd treatment cycle (T2) and the end of the 4th treatment cycle (T4). Imaging modalities were standardized to ensure consistency in response assessment. Contrast-enhanced computed tomography (CT) of the chest, abdomen, and pelvis served as the primary modality; magnetic resonance imaging (MRI) was substituted for patients with contraindications to iodinated contrast agents (e.g., severe renal impairment, documented contrast allergy). Additionally, bone scans were mandatory for patients presenting with clinical signs suggestive of bone involvement such as localized bone pain or unexplained elevation of alkaline phosphatase or those with baseline bone lesions. Positron emission tomography-computed tomography (PET-CT) was reserved for cases where CT/MRI findings were indeterminate (e.g., lesions of unclear malignant potential) to confirm or rule out distant metastasis.

d. Hematological Assessment: Complete peripheral blood test data retrievable from EMRs and LIS, with blood samples collected at baseline (T0) and during treatment at least four follow-up points, post-first cycle (T1), post-second cycle (T2), post-third cycle (T3), and post-fourth cycle (T4). Regarding data completeness: among the 150 initially screened patients, only 3 (2%) were excluded due to missing hematological data. The final 99 enrolled patients had intact data at all time points (T0-T4), which is suitable for dynamic analysis;

e. Clinical Data: Comprehensive clinical information was available, including demographic data, disease-related information, treatment details, and follow-up records.

f. Baseline Functional Status: Patients had an ECOG (Eastern Cooperative Oncology Group) performance status ≤2, indicating sufficient tolerance for treatment.

The exclusion criteria were as follows:

a. Inability to undergo tumor response evaluation: Patients who could not undergo tumor response evaluation per the irRECIST criteria, including those who did not complete imaging assessments at specified times during treatment (e.g., patients with interrupted treatment and no follow-up imaging data).

b. Prior or concurrent malignancies: Patients with prior or concurrent diagnoses of other malignancies, even if the lesions were completely resolved (CR).

c. Severe comorbidities: Patients with severe comorbidities that could affect the efficacy of immunotherapy or the study parameters, including but not limited to autoimmune diseases (e.g., systemic lupus erythematosus, rheumatoid arthritis), active tuberculosis, severe infections (e.g., sepsis, AIDS), or major organ failure (e.g., cardiac insufficiency, renal failure).

d. Medications interfering with treatment: Patients who used medications that could significantly interfere with the treatment effects during PD-1 therapy, such as high-dose corticosteroids (equivalent to >10 mg/day prednisone for over two weeks) or other immunosuppressants (e.g., cyclosporine, tacrolimus).

e. Pregnancy or lactation: Pregnant or lactating women.

f. Severe hepatic or renal disease: Patients with severe hepatic or renal disease, including significant hepatic impairment (ALT/AST levels exceeding five times the upper limit of normal, or total bilirubin levels exceeding three times the upper limit of normal) or severe renal impairment (eGFR <30 mL/min/1.73m^2^).

g. Other factors affecting the study: Patients with poor data integrity or missing key clinical/biological records (e.g., imaging studies, pathology reports, treatment records). Patients who discontinued treatment or were lost to follow-up without efficacy data were also excluded.

This study was approved by the Medical Ethics Committee of the First Affiliated Hospital of Zhengzhou University (Approval No.: 2024-KY-2269-00).

### 2.2 Demographic data

Demographic and baseline clinical characteristics were collected from internal clinical records. These included age, sex, smoking history, tumor histological type, treatment line, treatment protocol, and Ki-67 proliferation index.

### 2.3 Peripheral blood markers

Hematological data were collected at five time points: baseline (T0, before PD-1 therapy), after the first treatment cycle (T1), second cycle (T2), third cycle (T3), and fourth cycle (T4). Peripheral blood markers included red blood cells, lymphocytes, monocytes, neutrophils, platelets, and serum albumin. Derived markers included:

Neutrophil-to-lymphocyte ratio (NLR): calculated as the neutrophil count divided by the lymphocyte count.

Platelet-to-lymphocyte ratio (PLR): calculated as the platelet count divided by the lymphocyte count.

Lymphocyte-to-monocyte ratio (LMR): calculated as the lymphocyte count divided by the monocyte count.

Systemic immune-inflammation index (SII): calculated as platelet × neutrophil/lymphocyte.

Prognostic nutritional index (PNI): calculated as serum albumin level (g/L) + 5 × lymphocyte count (×10^9/L).

### 2.4 Treatment response evaluation

Treatment efficacy was assessed using the immune-related Response Evaluation Criteria in Solid Tumors (irRECIST) through imaging techniques such as CT, MRI, bone scans, or PET-CT at T0,T2 and T4. To avoid misclassification of pseudoprogression (a distinct immune-related response), patients initially meeting PD criteria per irRECIST were re-evaluated via confirmatory imaging (same modality as baseline) 4–8 weeks later. Those with stable/reduced tumor burden and no clinical deterioration (ECOG PS stable/improved) were classified as pseudoprogression and excluded from the non-benefit group (PD); only patients with persistent tumor growth or new lesions were confirmed as true PD and included in the non-benefit group. Patients were categorized as follows:

Clinical benefit group: patients with CR(complete response), PR(partial response), or SD(stable disease).

Non-benefit group: patients with PD(progressive disease).

### 2.5 Quality control measures

A “double data entry + cross-verification” model was adopted to ensure accuracy: two independent researchers extracted data from EMRs and LIS separately, established independent databases, and cross-checked for discrepancies. Discrepancies were resolved by reviewing original test reports, treatment records, or clinical notes before updating the database. All flagged values were verified against EMRs and LIS raw data, confirming they represented genuine clinical variations (e.g., transiently elevated NLR due to acute upper respiratory tract infection) with no entry errors, so were retained.

### 2.6 Statistical analysis

We performed statistical analyses using R software (version 4.3.0), and graphs were generated with GraphPad Prism (version 10.4). Baseline characteristics between the clinical benefit and non-benefit groups were compared using chi-square tests. To assess changes in hematological markers across four treatment cycles, we used two-way repeated measures analysis of variance (rmANOVA) to account for repeated data. Greenhouse-Geisser correction was applied when necessary. For comparisons at specific time points, t-tests were used with Benjamini-Hochberg false discovery rate (FDR) correction (m=30, with FDR controlled at 5%) to adjust for multiple comparisons. Stepwise logistic regression was used to identify predictors of treatment efficacy, with entry and removal criteria set at P<0.05 and P>0.10, respectively. We checked for multicollinearity among predictor variables using the variance inflation factor (VIF), and no significant collinearity (VIF < 10) was found. Receiver operating characteristic (ROC) curves were used to evaluate the prognostic value of hematological markers, and the optimal cutoff values were determined by the Youden Index (sensitivity + specificity − 1). A P value of less than 0.05 was considered statistically significant.

It is important to note that the model used in this study has not been validated internally or externally. The reported AUC is preliminary and should be considered exploratory, as no calibration curves were included in the analysis.

## 3 Result

**Figure 1:**
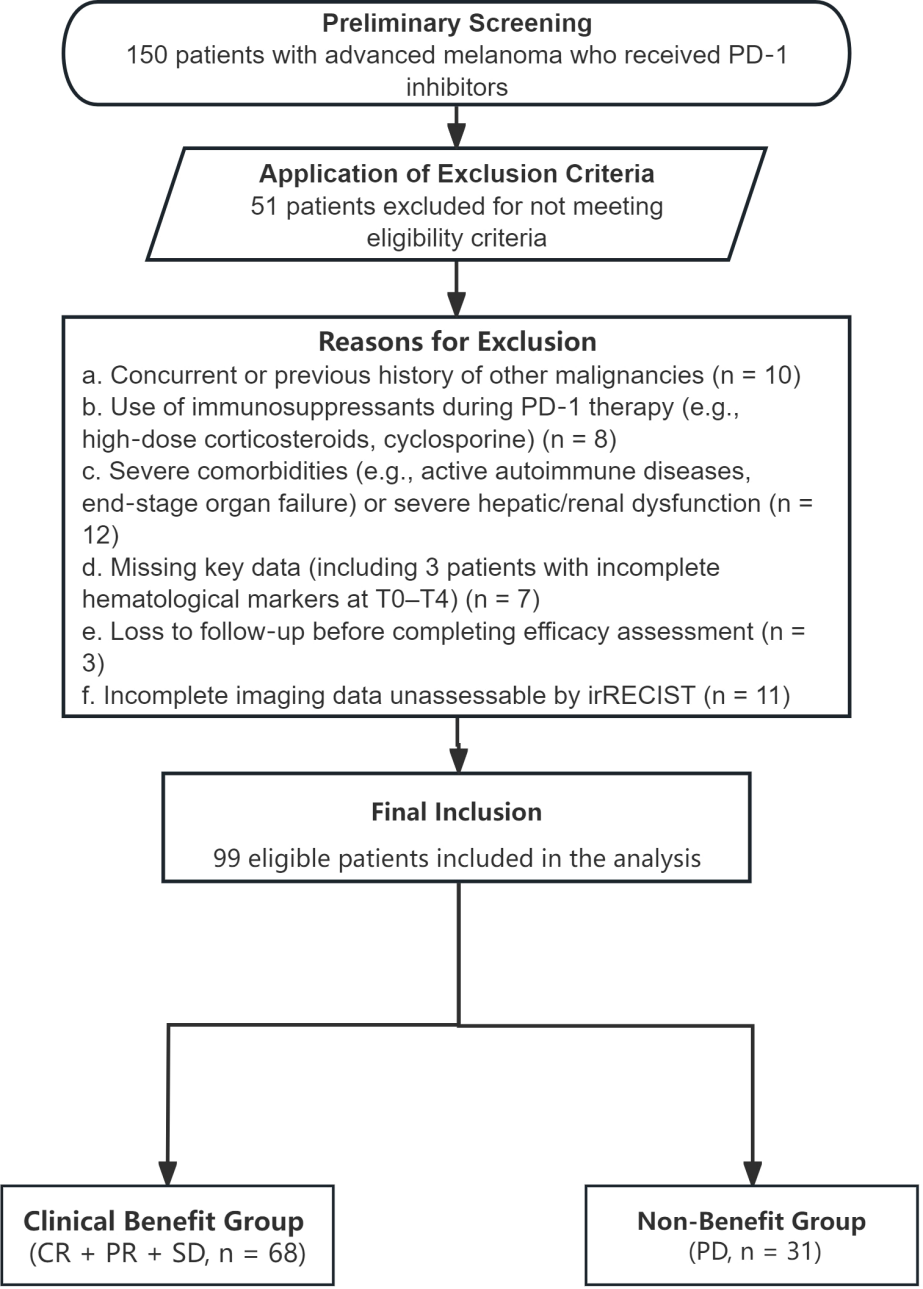
Flowchart of the patient selection process.

A total of 150 patients were initially screened from EMRs, and 99 were finally enrolled after applying inclusion/exclusion criteria (see **Figure 1** for patient selection flowchart). Among the enrolled patients: 42 were males and 57 were females. Ninety-three patients (93.9%) were aged ≤70 years, while six patients (6.1%) were aged >70 years. Seventeen patients (17.2%) had a history of smoking. Based on histological classification combined with anatomical origin and biological distinctiveness, 35 cases were identified as cutaneous melanoma(non-acral), 29 as acral melanoma, 29 as mucosal melanoma, and 6 as uveal melanoma. Ki-67 expression was determined through immunohistochemistry. The detailed clinical and pathological characteristics of all patients are presented in **Table 1**.

**Table 1:** Baseline clinicopathological characteristics of patients with advanced malignant melanoma.

Variables	Total (n = 99)	Non-benefit (n = 31)	Clinical benefit (n = 68)	*P*
Sex, n(%)				0.345
Male	42 (42.42)	11 (35.48)	31 (45.59)	
Female	57 (57.58)	20 (64.5)	37 (54.41)	
Age category, n(%)				0.731
≤70	93 (93.94)	30 (96.77)	63 (92.65)	
>70	6 (6.06)	1 (3.23)	5 (7.35)	
TNM Stage, n(%)				0.822
Unresectable Stage 3 (T3-4N2c-3M0)	31 (31.31)	10 (35.71)	21 (29.58)	
Stage 4 (T3-4N1-2M1a-b)	44 (44.44)	12 (42.86)	32 (45.07)	
Stage 4 (T4N2-3M1c-d)	24 (24.24)	6 (21.43)	18 (25.35)	
Smoking history, n(%)				0.853
No	82 (82.83)	26 (83.87)	56 (82.35)	
Yes	17 (17.53)	5 (16.67)	12 (17.91)	
Line of treatment, n(%)				0.068
First-line	61 (61.62)	15 (48.39)	46 (67.65)	
Multi-line	38 (38.38)	16 (51.61)	22 (32.35)	
Treatment strategy, n(%)				0.630
Immuno only	41 (41.41)	15 (48.39)	26 (38.24)	
Chemo+Immuno	39 (39.39)	11 (35.48)	28 (41.18)	
Targeted+ Immuno	19 (19.19)	5 (16.13)	14 (20.59)	
Subtype, n(%)				0.784
Cutaneous(non-acral)	35 (35.35)	13 (41.94)	22 (32.35)	
Uveal	6 (6.06)	1 (3.23)	5 (7.35)	
Mucosal	29 (29.29)	9 (29.03)	20 (29.41)	
Acral	29 (29.29)	8 (25.81)	21 (30.88)	
Ki-67 group, n(%)				<.001
0<Ki-67 ≤ 30%	44 (44.44)	4 (12.90)	40 (58.82)	
30% <Ki-67 ≤ 60%	48 (48.48)	24 (77.42)	24 (35.29)	
60% <Ki-67 ≤ 90%	7 (7.07)	3 (9.68)	4 (5.88)	

χ²: Chi-square test, -: Fisher exact.

There were no significant differences in age, sex, TNM stage (P = 0.822), smoking history, or histological subtypes between the clinical benefit group and the non-benefit group. However, Ki-67 expression significantly influenced treatment efficacy, with higher Ki-67 expression associated with poorer outcomes (P < 0.001).

Among the 99 patients, 61 had not received prior treatment before initiating PD-1 therapy, while 38 had undergone previous treatments. Specifically, 39 patients received PD-1 monotherapy, 41 received PD-1 immunotherapy combined with chemotherapy, and 19 received PD-1 immunotherapy combined with targeted therapy. No statistically significant differences were observed among these three treatment regimens (P = 0.63).

### 3.1 Hematological characteristics and dynamic changes between response and non-response groups

#### 3.1.1 Hematological characteristics after four cycles of PD-1 or PD-1 combination therapy

To explore potential indicators influencing the efficacy of immunotherapy, we first analyzed differences in hematological parameters between the clinical benefit and non-benefit groups after four cycles of PD-1-based therapy. To avoid false positive results caused by multiple comparisons, the Benjamini-Hochberg method was used for false discovery rate (FDR) correction (total number of tests m=30, false discovery rate controlled within 5%). After FDR correction, 15 indicators remained statistically significant between the two groups: T0ALB (P < 0.001), T0PNI (P < 0.001), T1NLR (P = 0.012), T1PLR (P < 0.001), T1LMR (P = 0.036), T1SII (P = 0.012), T1ALB (P < 0.001), T1PNI (P < 0.001), T2NLR (P < 0.001), T2SII (P < 0.001), T2ALB (P < 0.001), T2PNI (P < 0.001), T3PLR (P = 0.012), T3PNI (P = 0.009), and T4ALB (P = 0.030). No statistically significant differences were found in other indicators between the two groups (**Table 2**).

**Table 2:** Intergroup differences in dynamic changes of hematological indicators between the clinical benefit and non-benefit groups over four treatment cycles.

Sample	Estimate1	Estimate2	P value	FDR-adjusted P	Significance
T0NLR	2.822	2.582	0.737	0.737	NS
T0PLR	181.887	155.785	0.335	0.335	NS
T0LMR	4.011	4.493	0.235	0.235	NS
T0SII	706.084	662.405	0.834	0.834	NS
T0ALB(g/L)	39.355	42.366	<.001	<0.001	***
T0PNI	47.182	51.511	<.001	<0.001	***
T1NLR	2.969	2.034	0.004	0.012	**
T1PLR	219.483	139.409	<.001	<0.001	***
T1LMR	3.246	4.481	0.028	0.036	*
T1SII	723.964	454.596	0.008	0.012	**
T1ALB(g/L)	39.313	42.462	<.001	<0.001	***
T1PNI	46.221	50.979	<.001	<0.001	***
T2NLR	4.575	1.963	<.001	<0.001	***
T2PLR	178.993	137.396	0.033	0.062	NS
T2LMR	5.01	3.973	0.315	0.315	NS
T2SII	1029.693	429.166	<.001	<0.001	***
T2ALB(g/L)	38.829	42.154	<.001	<0.001	***
T2PNI	46.244	50.514	<.001	<0.001	***
T3NLR	2.648	2.348	0.397	0.397	NS
T3PLR	192.248	142.619	0.008	0.012	*
T3LMR	3.367	4.497	0.105	0.105	NS
T3SII	608.843	507.619	0.27	0.270	NS
T3ALB(g/L)	40.297	41.604	0.058	0.069	NS
T3PNI	47.327	49.819	0.006	0.009	**
T4NLR	2.782	2.236	0.233	0.233	NS
T4PLR	200.625	152.894	0.098	0.110	NS
T4LMR	3.905	4.184	0.58	0.580	NS
T4SII	654.942	464.04	0.123	0.132	NS
T4ALB(g/L)	40.329	41.826	0.023	0.030	*
T4PNI	48.327	49.89	0.205	0.205	NS


Table legend: Estimate 1 and Estimate 2 represent the non-benefit group and clinical benefit group, respectively, at each time point (T0–T4).

* p<0.05 ** p<0.01 ***p<0.001; NLR, neutrophil-to-lymphocyte ratio; PLR, platelet-to-lymphocyte ratio; LMR, lymphocyte-to-monocyte ratio; SII, systemic immune-inflammation index; ALB, albumin; PNI, prognostic nutritional index.

**Figure 2:**
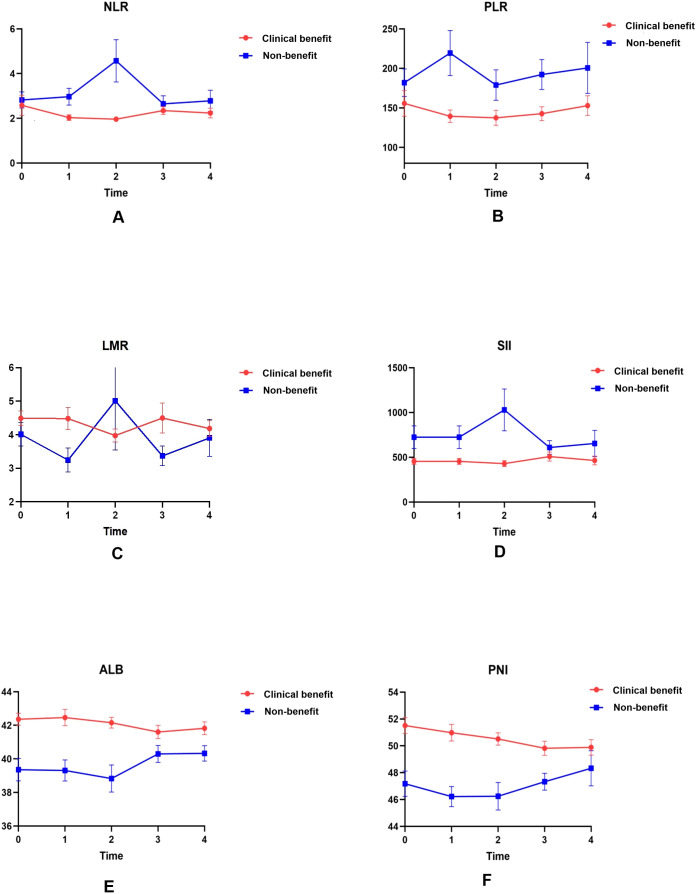
Dynamic changes in serological indicators in the clinical benefit and non-benefit groups over four treatment cycles. Absolute counts of NLR **(A)**, PLR **(B)**, LMR **(C)**, SII **(D)**, ALB **(E)**, and PNI **(F)** are plotted as line charts at baseline (T0) and after each of the first three cycles of PD-1 therapy (T1–T3).

By comparing the specific differences (**Table 2**, **Figure 2**), it was observed that in the non-benefit group, the mean values of T0ALB, T0PNI, T1LMR, T1PNI, T2ALB, T2PNI, and T4ALB were significantly lower than those in the clinical benefit group. Conversely, the mean values of T1NLR, T1PLR, T1SII, T2NLR, T2SII, and T3PLR were significantly higher in the non-benefit group compared to the clinical benefit group.

#### 3.1.2 Dynamic changes and differences in hematological indicators between clinical benefit and non-benefit groups

**Figure 3:**
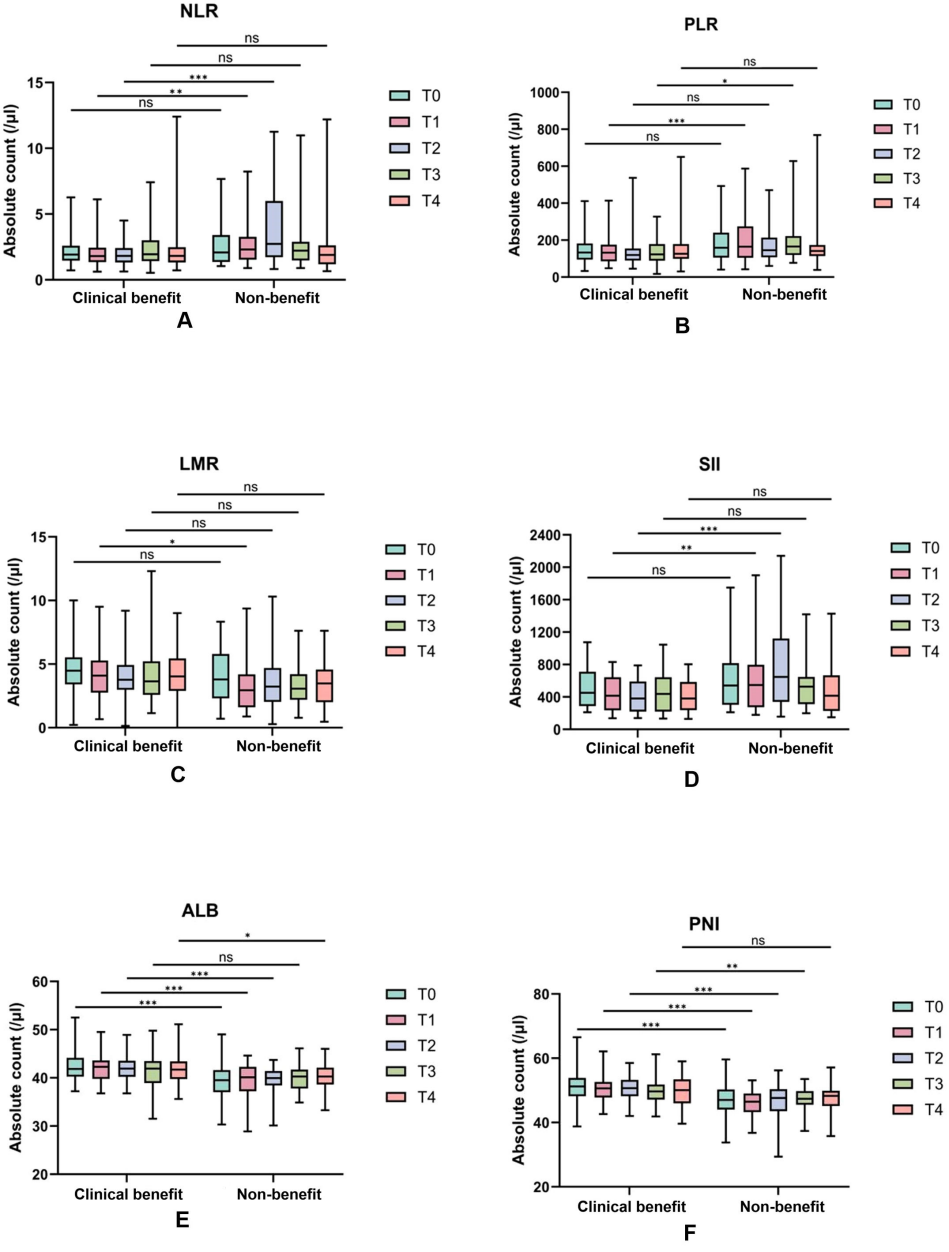
Dynamic changes of serological indexes in the efficacy group and the non-response group during the four treatment cycles. Absolute counts of NLR **(A)**, PLR **(B)**, LMR **(C)**, SII **(D)**, ALB **(E)**, and PNI **(F)** in the clinical benefit and non-benenefit groups over four consecutive treatment cycles are represented as boxplots. The t-test was used to analyze the differences between groups. *, p < 0.05;**, p < 0.01;,P<0.001; NS, no significant difference.

Given that single time-point data may be influenced by multiple external factors, current research emphasizes the dynamic changes of these indicators, a core analytical novelty designed to enhance benefit prediction accuracy. Line charts visualized mean values across baseline (T0) and four treatment cycles (T1–T4), while complementary boxplots depicted medians, interquartile ranges, and intergroup differences (addressing limitations of mean values in capturing data variability; **Figures 2** and **3**). Distinct dynamic patterns differentiating benefit patients from non-benefit patients emerged, with the most clinically actionable signals concentrated at T1 and T2 (early treatment time points):

NLR and SII: clinical benefit group maintained consistently lower NLR values than non-benefit group throughout treatment. Non-benefit group exhibited a marked NLR elevation peaking at T2 whereas clinical benefit group showed minimal fluctuations (**Figure 2A**). SII followed a parallel trend (**Figure 2D**), with the T2 peak in non-benefit group representing a potential early warning of suboptimal treatment response.

PLR: Non-clinical benefit group had persistently higher PLR values, which peaked at T1 before declining (**Figure 2B**). This T1 peak identifies the earliest discriminative time point for rapid treatment efficacy assessment in clinical practice.

LMR: Clinical benefit group displayed an initial LMR increase peaking at T2, whereas non-responders experienced a corresponding decrease to their lowest T2 values (**Figure 2C**). This opposing T2 trend reinforces the utility of early dynamic shifts for response stratification.

ALB and PNI: In contrast, ALB and PNI levels remained consistently higher in clinical benefit group than non-benefit group, with minimal temporal variability in both groups. This stability suggests preserved nutritional and immune status may serve as a baseline protective factor for favorable immunotherapy outcomes.

Both line charts and boxplots confirmed these distinct dynamic profiles, with significant intergroup differences annotated at key time points. Notably, these early-cycle dynamic changes fill a critical gap in PD-1 immunotherapy monitoring by providing timely actionable insights for clinical decision-making, enabling proactive adjustment of treatment strategies before overt disease progression.

### 3.2 Prognostic factors for predicting the efficacy of immunotherapy

#### 3.2.1 Prognostic impact of baseline hematological indicators on PD-1 immunotherapy

Being able to predict the efficacy of immunotherapy prior to its initiation would be highly ideal and valuable. Given that we have previously screened baseline peripheral blood indicators with significant intergroup differences via t-tests (supplemented with FDR correction) in the preceding section, we first evaluated these indicators for multicollinearity using the variance inflation factor (VIF), indicating no severe collinearity. On this basis, this study further explored the prognostic value of these baseline indicators for immunotherapy outcomes, using the response group (1) and non-response group (0) as the endpoint.

Univariate Logistic Regression: Baseline indicators that showed significant differences, such as T0ALB and T0PNI, were included in univariate logistic regression analysis. The results revealed that baseline T0ALB (P < 0.001) and T0PNI (P < 0.001) were prognostic factors for the efficacy of PD-1 immunotherapy in patients with malignant melanoma.

Multivariate Logistic Regression: Stepwise multivariate logistic regression (bidirectional selection) was performed for further analysis, and baseline T0ALB was identified as an independent prognostic factor for the efficacy of PD-1 immunotherapy (**Tables 3** and **4**).

**Table 3:** Univariate and multivariate logistic regression analyses of baseline hematological indicators for predicting clinical benefit from PD-1 immunotherapy in patients with advanced malignant melanoma.

Variables	Univariate					Multivariate				
	β	S.E	Z	P	OR(95%CI)	β	S.E	Z	P	OR(95%CI)
T0ALB	0.33	0.09	3.56	**<.001**	1.39 (1.16 ~ 1.66)	0.33	0.09	3.56	<.001	1.39 (1.16 ~ 1.66)
T0PNI	0.19	0.05	3.42	**<.001**	1.20 (1.08 ~ 1.34)					

Bold values denote statistically significant associations (P < 0.05) between hematological indicators and clinical benefit from PD-1 immunotherapy.

**Table 4:** Receiver operating characteristic (ROC) metrics for individual hematological indicators and multivariable models in predicting clinical benefit from PD-1 immunotherapy in patients with advanced malignant melanoma.

Predictor/Model	AUC (95%CI)	Sensitivity (95%CI)	Specificity (95%CI)	Cutoff value	Cutoff definition
T0ALB(g/L)	0.75 (0.64-0.86)	0.74(0.59- 0.90)	0.69(0.58- 0.80)	41.05	Optimal threshold of T0ALB for clinical benefit prediction (Youden Index)
T1PLR	0.66 (0.54–0.78)	0.71 (0.55–0.87)	0.57 (0.46–0.69)	141.456	Optimal threshold of T1PLR for clinical benefit prediction (Youden Index)
T1ALB(g/L)	0.72 (0.61–0.83)	0.74 (0.59–0.90)	0.65 (0.53–0.76)	41.3 g/L	Optimal threshold of T1ALB for clinical benefit prediction (Youden Index)
T2NLR	0.70 (0.58–0.82)	0.55 (0.37–0.72)	0.82 (0.73–0.91)	2.704	Optimal threshold of T2NLR for clinical benefit prediction (Youden Index)
Combined model (T1PLR+T1ALB+T2NLR)	0.89 (0.82–0.97)	0.90 (0.80–1.00)	0.79 (0.70–0.89)	0.769	Statistical probability threshold (Youden Index); not for direct clinical use

**Figure 4:**
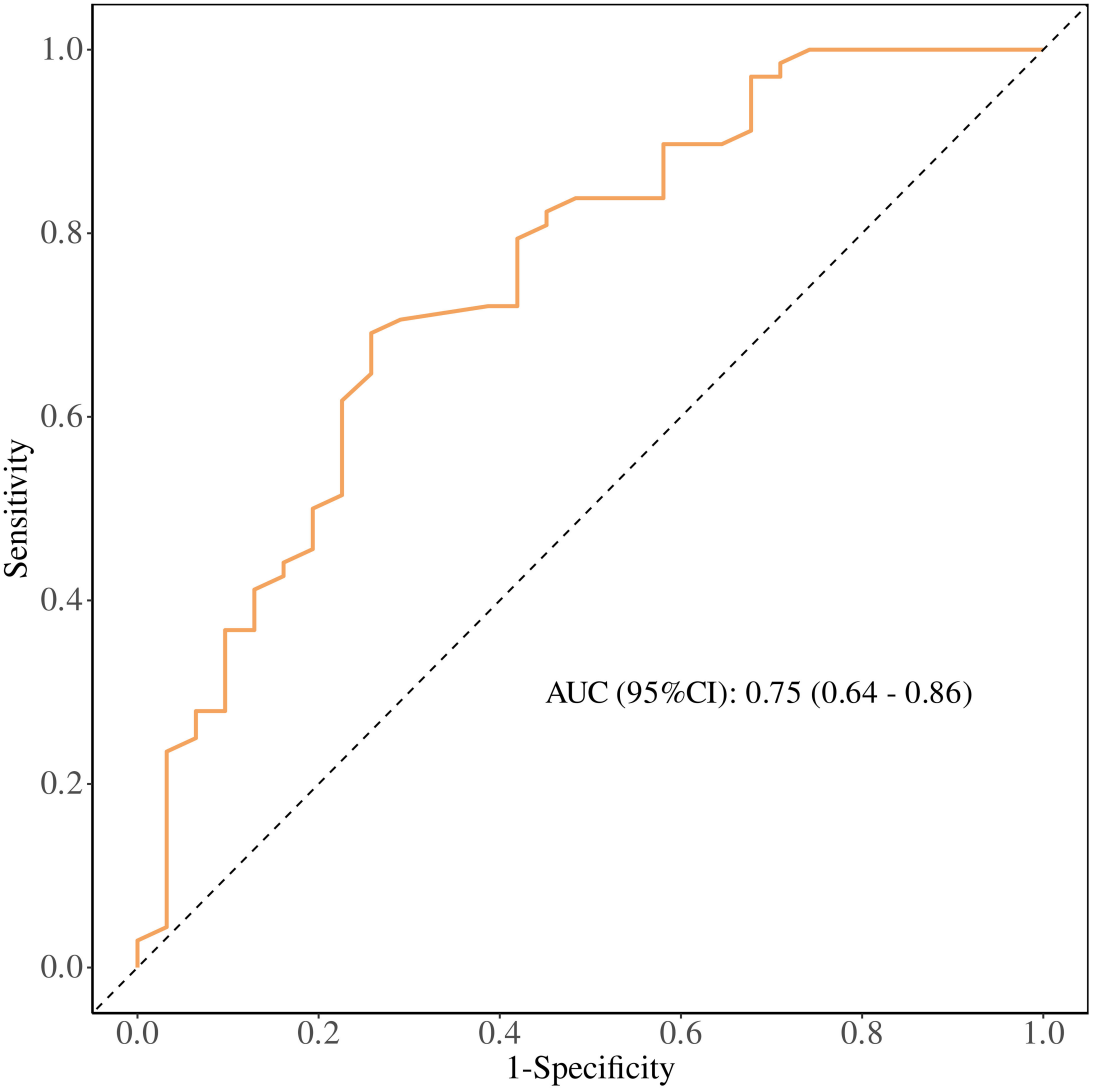
Receiver operating characteristic (ROC) curve of baseline serum albumin (T0 ALB) for predicting clinical benefit from PD-1 immunotherapy in patients with advanced malignant melanoma. The area under the curve (AUC) with 95% confidence interval and the Youden-derived optimal cut-off value are shown.

ROC Curve Analysis: The performance of the regression model was evaluated using a receiver operating characteristic (ROC) curve. The area under the curve (AUC) was 0.75, with a sensitivity of 0.74, specificity of 0.69, and a cutoff value of 41.05 g/L. Patients with baseline T0ALB ≥ 41.05 g/L (OR = 1.39, 95% CI: 1.16–1.66, P < 0.001) were more likely to benefit from immunotherapy. Increased baseline T0ALB levels were associated with improved immunotherapy efficacy. (**Figure 4**).

#### 3.2.2 Prognostic impact of early treatment hematological indicators on PD-1 immunotherapy

While baseline data provide valuable insights, they are influenced by multiple factors. Early changes in peripheral blood indicators (i.e., after 1–2 treatment cycles) may more directly correlate with immunotherapy response. Given that we have previously screened T1 and T2 peripheral blood indicators with significant intergroup differences via t-tests (supplemented with FDR correction) in the preceding section, we first evaluated these indicators for multicollinearity using the variance inflation factor (VIF), indicating no severe collinearity. On this basis, this study aimed to explore the prognostic impact of early treatment data on immunotherapy efficacy.

Univariate Logistic Regression: Indicators showing differences at treatment cycles T1 and T2, including NLR, PLR, LMR, SII, ALB, and PNI, were included in univariate analysis. This analysis revealed that T1NLR, T1PLR, T1LMR, T1SII, T1ALB, T1PNI, T2NLR, T2PLR, T2SII, T2ALB, and T2PNI were prognostic factors for the efficacy of PD-1 immunotherapy in malignant melanoma (**Tables 4** and **5**).

**Table 5:** Univariate and multivariate logistic regression analyses of early-treatment (T1/T2) hematological indicators for predicting PD-1 immunotherapy efficacy.

Variables	Univariate		Multivariate	
	*P*	OR (95%CI)	*P*	OR (95%CI)
T1NLR	**0.009**	0.67 (0.50 ~ 0.91)		
T1PLR	**0.004**	0.99 (0.99 ~ 0.99)	**0.016**	0.99 (0.99 ~ 0.99)
T1LMR	**0.026**	1.33 (1.03 ~ 1.70)		
T1SII	**0.021**	0.99 (0.99 ~ 0.99)		
T1PNI	**<.001**	1.31 (1.15 ~ 1.50)		
T1ALB(g/L)	**<.001**	1.40 (1.16 ~ 1.69)	**0.008**	1.44 (1.10 ~ 1.89)
T2NLR	**<.001**	0.53 (0.37 ~ 0.77)	**0.006**	0.54 (0.35 ~ 0.84)
T2SII	**0.003**	0.99 (0.99 ~ 0.99)		
T2PNI	**<.001**	1.23 (1.10 ~ 1.39)		
T2ALB(g/L)	**<.001**	1.44 (1.17 ~ 1.78)	0.152	1.23 (0.93 ~ 1.63)

OR, Odds Ratio, CI, Confidence Interval. Bold values denote statistically significant associations (P < 0.05) between hematological indicators and clinical benefit from PD-1 immunotherapy.

Multivariate Logistic Regression: Stepwise multivariate logistic regression identified T1PLR, T1ALB, and T2NLR as independent predictors of immunotherapy efficacy.

ROC Curve Analysis: The performance of the regression model was evaluated using a receiver operating characteristic (ROC) curve, with an area under the curve (AUC) of 0.89, sensitivity of 0.90, and specificity of 0.79. To further explore the prognostic ability of T1PLR, T1ALB, and T2NLR for immunotherapy efficacy, individual ROC curves were plotted. The AUCs for these markers were:

T1PLR: 0.66, cutoff value 141.456.

T1ALB: 0.72, cutoff value 41.3 g/L.

T2NLR: 0.70, cutoff value 2.704.

Significant Predictors: Patients with:

T1PLR ≤ 141.456 (OR = 0.99, 95% CI: 0.99–0.99, P = 0.016).

T1ALB ≥ 41.3 g/L (OR = 1.44, 95% CI: 1.10–1.89, P = 0.008).

**Figure 5:**
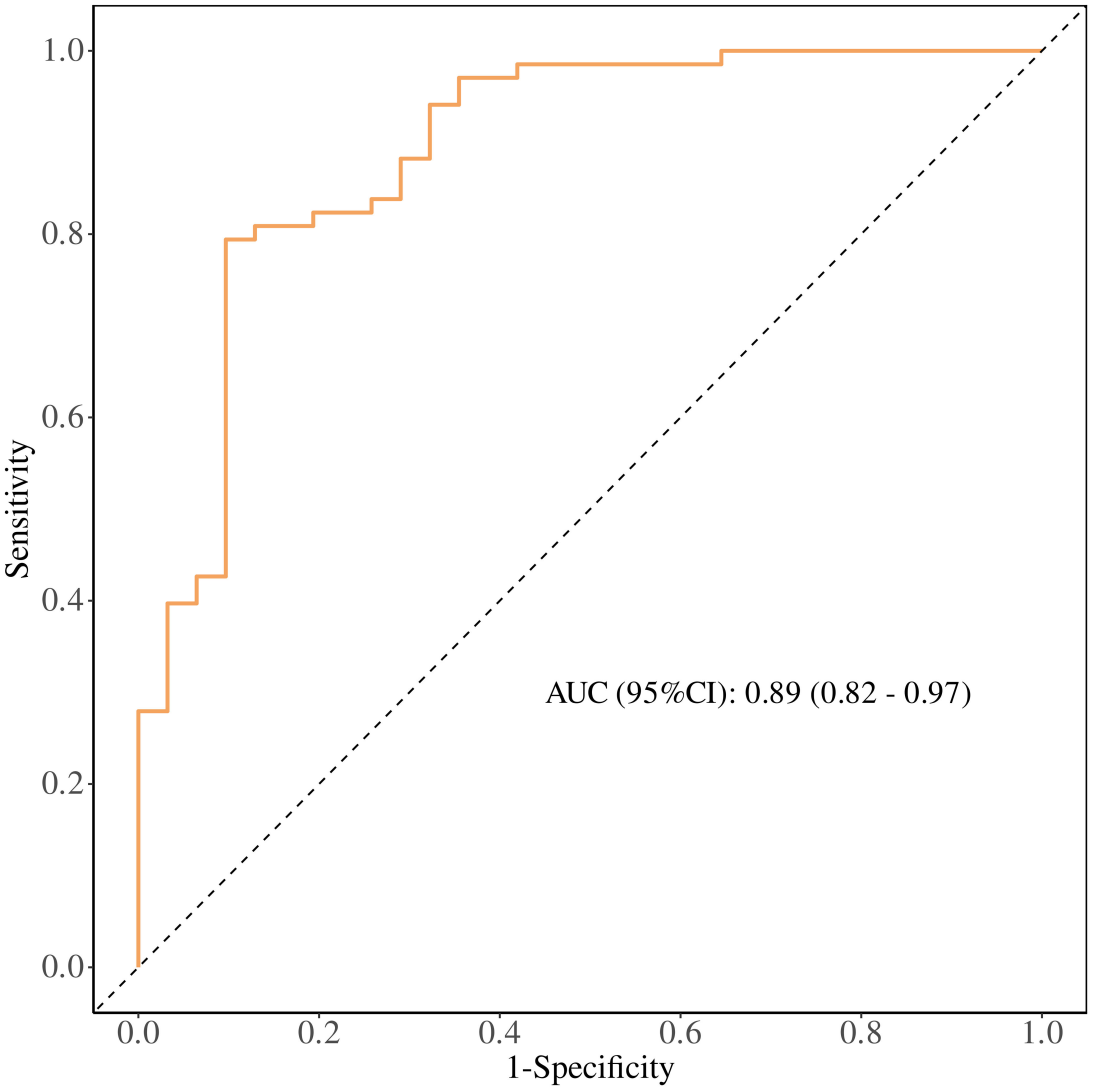
Receiver operating characteristic (ROC) curve of the multivariable model combining early treatment indicators (T1 PLR, T1 ALB, and T2 NLR) for predicting clinical benefit from PD-1 immunotherapy in patients with advanced malignant melanoma. The area under the curve (AUC) with 95% confidence interval and the Youden-derived optimal cut-off value are shown.

T2NLR ≤ 2.704 (OR = 0.54, 95% CI: 0.35–0.84, P = 0.06).

were more likely to benefit from immunotherapy. Early increases in ALB, as well as decreases in PLR and NLR, were associated with better immunotherapy outcomes. (**Figure 5**).

## 4 Discussion

This study found that the dynamic changes in hematological indicators such as NLR, PLR, and ALB have significant prognostic value in patients with advanced malignant melanoma undergoing PD-1 immunotherapy. Specifically, patients with early treatment cycle values of PLR < 141.456, ALB ≥ 41.3 g/L, and NLR <2.703 were more likely to benefit from treatment, while the combined prognostic model integrating these three indicators achieved an AUC of 0.89, confirming its potential as a practical clinical tool for treatment stratification.

Our findings build upon and extend the growing body of evidence linking hematological markers to immunotherapy outcomes in melanoma. Consistent with prior research, NLR and PLR, well-recognized surrogates of systemic inflammation and tumor microenvironment immunosuppression were identified as reliable predictors of PD-1 inhibitor response (35, 36). For instance, a recent study evaluating dynamic inflammatory markers in melanoma patients treated with anti-PD-1 monotherapy or ipilimumab–nivolumab combinations reported that early-cycle NLR and PLR fluctuations correlated with treatment response and survival, aligning with our observation of T1 PLR peaks and T2 NLR elevations in non-benefit patients (37). Complementing these findings, a recent prospective study demonstrated that dynamic changes in C-reactive protein (CRP), another key systemic inflammatory marker, served as a robust prognostic indicator in melanoma patients receiving immunotherapy, further supporting the clinical value of serial inflammatory marker monitoring for predicting treatment outcomes (38).

Regarding serum albumin (ALB), our data confirm its robust prognostic role in melanoma immunotherapy response: levels remained consistently higher in the clinical benefit group across all time points, with T1 ALB ≥ 41.3 g/L independently associated with favorable treatment outcomes. While prior investigations have explored immunonutritional markers—including albumin—in melanoma patients undergoing immunotherapy (39), these studies have primarily centered on baseline measurements or single-timepoint assessments. Our work builds on this foundation by offering complementary and clinically actionable insights through two key observations. First, we demonstrate the sustained stability of ALB throughout the early treatment course (T0–T2) in responders—a distinct pattern that differentiates patients with adaptive therapeutic responses from those with suboptimal outcomes. Second, we identify the synergistic prognostic efficacy of ALB when integrated with early-cycle inflammatory markers (T1PLR and T2NLR); this combination yielded a superior prognostic model (AUC = 0.89), outperforming single-marker assessments or baseline-only approaches reported in previous literature. This distinction is clinically meaningful given the well-established links between nutritional status, immune cell function, and immunotherapy efficacy. Preserved ALB stability during early treatment may reflect intact systemic immune competence and reduced tumor-induced catabolism—two important determinants of effective anti-tumor immunity that extend beyond the static nutritional assessment provided by baseline albumin measurements alone.

The biological mechanisms underlying these observations are consistent with established immunopathogenic pathways in melanoma. NLR (40) and PLR (41), as inflammatory markers, reflect the immune status of the tumor microenvironment and systemic inflammatory response. High NLR and PLR levels may indicate an immunosuppressive environment, allowing tumor cells to evade immune surveillance, thereby resulting in poor treatment outcomes. Specifically, neutrophils (42, 43)promote tumor growth and metastasis through the secretion of pro-inflammatory factors, while a reduced lymphocyte count reflects weakened anti-tumor immunity. Platelets play a crucial role in tumor metastasis and immune suppression (44), as they secrete pro-inflammatory factors such as CXCL1 and CXCL4, facilitating leukocyte recruitment and promoting tumorigenesis and progression (45, 46). ALB, as a nutritional marker, represents the overall nutritional status and stress response of patients. A good nutritional status helps maintain immune cell function and enhances treatment response (47, 48). The dynamic changes in these indicators can reflect patients’ physiological status during treatment in real-time, providing a basis for individualized treatment.

The findings of this study provide new directions for precision medicine in patients with advanced malignant melanoma. By dynamically monitoring NLR, PLR, and ALB, clinicians can identify patients who are less likely to respond to PD-1 immunotherapy early in the treatment course and adjust therapeutic regimens promptly, thereby improving overall treatment efficacy. Additionally, monitoring these hematological indicators is simple, cost-effective, and highly feasible for routine clinical practice. This not only aids in developing individualized treatment strategies but also reduces unnecessary treatment burden and improves patients’ quality of life.

Notably, our focus on early treatment dynamics (T1–T2) directly addresses a critical unmet clinical need in melanoma immunotherapy: distinguishing adaptive therapeutic responses from high-risk aberrant outcomes like hyperprogression (HPD) (49). HPD is a well-documented and clinically concerning phenomenon in ICI-treated patients, defined by rapid tumor growth and poor prognosis, an event that demands early identification to avoid futile treatment and enable timely intervention (50–51).

Consistent with findings from the study, HPD has been linked to dysregulated systemic inflammation and compromised nutritional status, characterized by elevated NLR and hypoalbuminemia. This aligns closely with our observations in the non-benefit group: pronounced T2 NLR surges, and persistently lower ALB levels throughout early treatment, patterns that mirror the HPD-associated biomarker signatures reported in prior literature (52, 53). While our study did not explicitly stratify cases by HPD status, the early dynamic shifts we identified—particularly the combination of aberrant inflammatory marker elevations (T1 PLR, T2 NLR) and reduced ALB stability—hold practical clinical relevance for HPD risk stratification. These readily measurable blood marker changes may serve as accessible warning signals for both suboptimal treatment responses and heightened HPD risk, allowing clinicians to adjust therapeutic strategies before overt disease progression.

Despite providing valuable insights, this study has several limitations. First, as a single-center retrospective study with a small sample size (response group: n = 68, non-response group: n = 31), the statistical robustness and generalizability of the results may be limited. Notably, the limited number of progression (PD) events (n=31) precluded adjusting for the baseline covariate(Ki-67, an established prognostic factor significantly associated with treatment response) in multivariate models. Adherence to statistical principles (10–15 events per covariate) was prioritized to mitigate overfitting, leading to unavoidable residual confounding. Second, inherent to retrospective studies, potential selection bias and residual confounding factors preclude comprehensive control of confounders—including the exclusion of patients with comorbidities (which likely yields a study population with more favorable prognosis than the general melanoma cohort), the exclusion of patients unable to complete tumor response assessments (who presumably had particularly poor outcomes), and unassessed subclinical mild infections or undiagnosed mild inflammatory disorders. These limitations may compromise the external validity of our findings. Third, the study included heterogeneous PD-1-based regimens (PD-1 monotherapy, chemo-immunotherapy, and targeted-immunotherapy combinations). While all regimens shared PD-1 inhibition as the core therapeutic component, the addition of cytotoxic chemotherapy (carboplatin plus paclitaxel) or targeted agents (apatinib) may have differentially influenced inflammatory and nutritional markers. These regimen-specific effects could not be fully disentangled due to the need to preserve statistical power—stratifying into separate subgroups by regimen type would have substantially reduced sample size per group, limiting the ability to detect meaningful associations between dynamic marker changes and clinical outcomes. Additionally, this study focused only on the dynamic changes in hematological indicators and did not incorporate molecular biology data to further explore their specific mechanisms of action. Future research is needed to validate these findings in larger, multi-center, prospective studies and to combine them with molecular biology and immunological mechanisms to fully understand the role of these indicators in immunotherapy. Lastly, a critical limitation concerns our prognostic models. The primary model (AUC = 0.89) was built via stepwise logistic regression, using 31 progression (PD) events and multiple candidate predictors—a “low event-to-variable ratio” that elevates overfitting risk (contrary to the 10–15 events/predictor guideline). No internal (e.g., bootstrapping) or external validation was performed to confirm stability or generalizability. Accordingly, the reported AUC = 0.89 is exploratory and hypothesis-generating, not a clinically actionable metric. Future work must refine the model in larger cohorts with adequate PD events, followed by rigorous validation, before clinical translation.

Future studies should focus on multi-center, large-sample prospective research to validate the prognostic value of NLR, PLR, and ALB in different types of immunotherapy. Exploring the combined use of these hematological indicators with other biomarkers could further improve prognostic accuracy. Mechanistic studies will also help elucidate the specific roles of these indicators in the immunotherapy response, providing a theoretical basis for developing new therapeutic strategies. Additionally, integrating multi-dimensional data to build dynamic prognostic models will help guide individualized treatment strategies more precisely, ultimately improving patient outcomes and prognosis.

## 5 Conclusion

In conclusion, this study confirms the importance of the dynamic changes in hematological indicators such as NLR, PLR, and ALB in predicting the efficacy of PD-1 immunotherapy in patients with advanced malignant melanoma. These indicators provide effective tools for clinical practice, aiding in the optimization of individualized treatment strategies and improving treatment response and prognosis for patients.

## Data Availability

The raw data supporting the conclusions of this article will be made available by the authors, without undue reservation.
