# Supplementary material for: Tracking temporal shifts of peripheral blood NLR, PLR, and ALB: a prognostic tool for PD-1 inhibitor efficacy in advanced malignant melanoma
Source: Front Oncol. 2026 Jan 5;15:1704359. doi: 10.3389/fonc.2025.1704359 (PMC12812586; doi:10.3389/fonc.2025.1704359)
Supplement: Supplementary file 1 [file Table1.docx]

Supplementary Table S1: Core rmANOVA Results for Dynamic Hematologic Parameters

| **Indicator** | Group × Time Interaction (F Value, DFn/Dfd) | Greenhouse-Geisser Corrected P Value | ε(Correction Coefficient) |
| --- | --- | --- | --- |
| **NLR** | 3.563 (4.000, 388.000) | 0.0072 | 0.7008 |
| **PLR** | 0.9292 (4.000, 388.000) | 0.4469 | 0.6988 |
| **LMR** | 2.091 (4.000, 388.000) | 0.0813 | 0.6750 |
| **SII** | 2.452 (4.000, 388.000) | 0.0456 | 0.6979 |
| **ALB** | 2.436 (4.000, 388.000) | 0.0468 | 0.9329 |
| **PNI** | 2.474 (4.000, 388.000) | 0.0440 | 0.8287 |
